# Depletion of Gut Microbiota Impairs Gut Barrier Function and Antiviral Immune Defense in the Liver

**DOI:** 10.3389/fimmu.2021.636803

**Published:** 2021-03-25

**Authors:** Weina Guo, Xin Zhou, Xiaoran Li, Qingfeng Zhu, Jing Peng, Bin Zhu, Xin Zheng, Yinping Lu, Dongliang Yang, Baoju Wang, Junzhong Wang

**Affiliations:** ^1^ Department of Infectious Diseases, Union Hospital, Tongji Medical College, Huazhong University of Science and Technology, Wuhan, China; ^2^ Department of Laboratory Medicine, Tongji Hospital, Tongji Medical College, Huazhong University of Science and Technology, Wuhan, China

**Keywords:** antibiotic therapy, gut microbiota, translocation, HBV infection, immune defense

## Abstract

Commensal gut microbiota protects the immune defense of extra-intestinal organs. Gut microbiota depletion by antibiotics can impair host antiviral immune responses and alter hepatitis B virus (HBV) infection outcomes. However, how gut microbiota modulates antiviral immune response in the liver remains unclear. Here, mice were treated with broad-spectrum antibiotics to deplete gut microbiota. Gut integrity was evaluated, and translocation of live commensal gut bacteria and their components into the liver was investigated. An HBV infection model was established to evaluate impairment of antiviral immune response in the liver after gut microbiota depletion. We found that gut microbiota depletion was associated with impairment of colon epithelial integrity, and live commensal gut microbiota could translocate to the liver. Further, T cell antiviral function in the liver was impaired, partially relying on enhanced PD-1 expression, and HBV immune clearance was hampered. In conclusion, gut microbiota depletion by antibiotics can impair gut barrier function and suppress T cell antiviral immune response in the liver.

## Introduction

The gut and liver closely interact with each other *via* the portal vein, biliary system, and systemic circulation, and both organs have significant physiological crosstalk, referred to as the gut-liver axis. Products/toxins and metabolites from bacteria in the gut enter the liver and metabolize/detoxicate in the liver. Meanwhile, many bioactive mediators, including primary bile acids, are synthesized in the liver and are transported to the intestine, thus influencing gut function. Increasing evidence indicates that the metabolites of the gut microbiota can affect immune response in the liver, and the gut-liver axis is considered pivotal in the progression of most liver diseases, including viral hepatitis, alcoholic and non-alcoholic fatty liver disease, auto-immune liver disease, liver cirrhosis, and hepatocellular carcinoma (1). Liver diseases can also affect commensal bacterial community and gut function. Hepatitis B virus (HBV) infection can alter gut microbiota composition and modulate the immune molecular expression in the intestine (2).

The commensal microbiota colonizes the gastrointestinal tract without inducing inflammation and injury in healthy hosts, because the gut barrier, which includes surface mucus, epithelial layer, and immune defense, can prevent the dissemination of microbes and toxic and luminal proinflammatory factors. In addition, the gut microbiota can modulate immune response in the gut by influencing mucus production (3). Antibiotic therapy can disrupt the gut microbiota and impair host defense immunity against some infectious diseases (4), allowing live commensal bacteria to break through the gut barrier and translocate to the mesenteric lymph node (MLN), resulting in low-level inflammation and worsened colitis (5). In an autoimmune mouse model, live *Enterococcus gallinarum* translocated from the gut to the liver and induced auto-antibodies. Monocolonization by *Enterococcus gallinarum* can down-regulate the expression of tight junction proteins, which in turn impair gut barrier function, indicating that gut microbiota composition can influence gut barrier function (6). However, little is known about the impact of antibiotic treatment on the gut barrier function and gut microbiota translocation to the liver with HBV infection.

Many chronic liver diseases are associated with gut microbiota dysbiosis. It is unclear whether the gut microbiota can translocate to the liver in chronic liver disease. In a mouse model of liver cirrhosis, the gut microbiota was found to translocate to the portal vein and liver and trigger tonic type I interferon signaling, which in turn led to interleukin (IL)−10 production in myeloid cells and impaired innate antibacterial immunity in the liver (7). Another study indicated that the gut microbiota contributes to HBV immunity and determines the outcome of HBV infection. Treatment with antibiotics impaired HBV-specific T cell response and resulted in persistent HBV infection in adult C3H/HeN mice (8). However, the mechanisms underlying microbiota translocation and modulation of HBV immunity remain unclear. In this study, we attempted to investigate the protective role of the gut microbiota in gut barrier function and antiviral immune response in the liver.

## Methods

### Animal Experiments

Four-week-old, male *C57BL/6* mice were purchased from Hunan Slack King Laboratory Animal Co., Ltd. (Changsha, China). The mice were housed under specific pathogen-free (SPF) conditions at the Animal Care Center of Tongji Medical College, Huazhong University of Science and Technology, Wuhan, China. This study was in accordance with the Guidelines for the Care and Use of Laboratory Animals of the National Institutes of Health, and the protocols were approved by the Institutional Animal Care and Use Committee at Tongji Medical College (Permit Number: S814).

To deplete the gut microbiota, mice were treated with a cocktail of broad-spectrum antibiotics (ABX; ampicillin 1 g/L, neomycin sulfate 1 g/L, metronidazole 1 g/L, vancomycin 0.5 g/L) in drinking water for 4 weeks as previously described (9, 10). To establish the HBV infection model, the HBV plasmid pSM2/HBV (provided by Dr. Hans Will, Heinrich-Pette-Institute, Hamburg, Germany), was hydrodynamically injected into the tail vein of the mice, as described in previous studies (2).

### Electron Microscopy Assay

The ultrastructure of colons was analyzed using scanning electron microscopy (SEM) and transmission electron microscopy (TEM), as previously described (11). For SEM, colon pieces were putter-coated with gold after briefly drying on tinfoil paper (SU-8010, Hitachi). For TEM, the colon pieces were fixed with 2.5% (v/v) glutaraldehyde at 4°C overnight, and ultrathin samples were sectioned and assayed using TEM assay (H-7000FA, Hitachi).

### Fecal Sample Collection and 16S rRNA Sequencing

Fecal pellets from the mice were collected and immediately frozen at -80°C. Bacterial 16S rRNA was amplified and extracted, pooled in equimolar ratios, and paired-end sequenced (2×300) on an Illumina MiSeq platform (Illumina, San Diego, USA), as previously reported (2).

### Determination of Molecules Related to Gut Barrier Function and 16S rRNA by qPCR

To determine the expression of molecules related to gut barrier function, total RNA was isolated from the colons using the TRIZOL reagent (Invitrogen, Carlsbad, CA, USA) according to the manufacturer’s instructions, and qPCR was performed using SYBR Green PCR Kit and QuantiTect Primers (Qiagen, Dusseldorf, NRW, Germany) on the CFX Connect™ Real-Time PCR Detection System (BIO-RAD, Hercules, CA, USA).

The bacterial DNA in fecal samples was extracted using a QIAamp Fast DNA Stool Mini Kit (Qiagen, Dusseldorf, NRW, Germany). The abundance of specific intestinal bacterial groups was measured using a qPCR Kit (TAKARA) and group-specific 16S rRNA gene primers, as previously described (12).

### Bacterial Culture and Identification

Liver from mice was homogenized using a sterile homogenizer, and liver suspensions were cultured on a blood agar plates at 37°C overnight. The colonies on the plates were identified using matrix-assisted laser desorption/ionization time-of-flight (MALDI-TOF) mass spectrometry, IVD-MALDI Biotyper (Bruker, Germany).

### Gut Permeability Assay

Mice were given 500 mg/kg of FITC–dextran (4.4 kDa; Sigma) by oral gavage, and were sacrificed 2 h later and sera and liver tissue was obtained. The fluorescence of the sera and liver homogenates was measured using a FLUOstar Optima Microplate Reader (BMG Labtech, Germany), as previously described (13).

### Bacterial Tracer Assay

*E. coli* (10^9^ CFU, colony-forming units) was stained with BacLight Green Bacterial Stain as recommended (Invitrogen), and then inoculated into mice *via* oral gavage. The mice were sacrificed 4 h later and livers were collected. Fluorescently labeled bacteria in liver tissue sections were analyzed using an Olympus microscope. The fluorescence intensity in the liver homogenate was assayed using a Multilabel Reader (Perkin EIMER, Singapore).

### Detection of Serological HBV Antigen and HBV DNA

HBsAg/HBeAg/HBsAb/HBeAb/HBcAb levels in sera were measured using ELISA kits (Kehua, Shanghai, China), according to the manufacturer’s instructions. HBV DNA copies were measured using a diagnostic HBV DNA kit (Sansure, Changsha, China) and real-time PCR, as previously reported (2).

### Immunohistochemistry (IHC), Immunofluorescence and Flow Cytometry Analysis

Tissue sections from liver and colon were analyzed using IHC and immunofluorescence. Intrahepatic lymphocytes were isolated and analyzed using flow cytometry. Details are provided in Supplementary Material and Methods.

### Statistical Analysis

Statistical analyses were performed using SPSS software version 12.0 (SPSS Inc, Chicago, IL, USA). Two-tailed unpaired Student’s t-tests and one-way ANOVA with Tukey’s multiple comparison tests were used to analyze the differences between two groups and among multiple groups, respectively. Results with a *p* value < 0.05 were considered statistically significant.

## Results

### Depletion of Gut Microbiota by Antibiotics Impaired the Colon Epithelial Integrity

**Figure 1:**
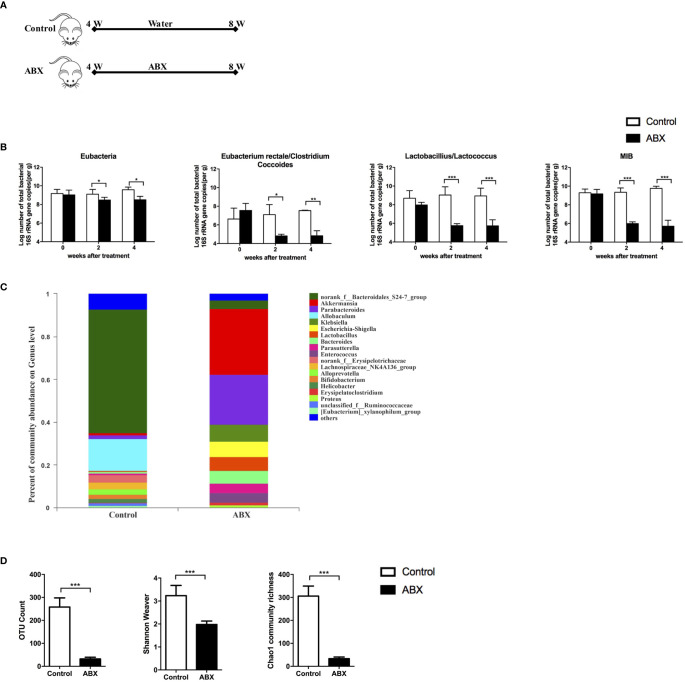
Quantitative analysis of the gut microbiota after ABX therapy. **(A)** Schedule of the therapy in mice fed with water or ABX for 4 weeks. **(B)** The abundance of specific commensal bacterial groups in fecal pellets measured using qPCR after ABX therapy. 16S rRNA sequencing from fecal pellets after ABX therapy. The composition of gut microbiota at genus-level **(C)**, the abundance and diversity of the gut microbiota **(D)** were assessed. n=10/group, *P < 0.05, **P < 0.01, ***P < 0.001.

To evaluate the effect of gut microbiota depletion on gut and liver function, 4−week-old *C57BL/6* mice were fed with water or ABX for 4 weeks (**Figure 1A**). Stool pellets were collected, and the gut microbiota composition was analyzed using qPCR and 16S RNA sequencing. Loads of eubacteria (all bacteria), *Eubacterium rectale/Clostridium coccoides*, *Lactobacillus/Lactococcus*, and mouse intestinal *Bacteroides* (MIB) decreased significantly at both 2 and 4 weeks post-ABX treatment compared to the control mice (**Figure 1B**). The percentages of gut microbiota at genus-level were altered after ABX treatment, especially *norank_f_Bacteroidales_S24-7_group* and *Allobaculum* decreased, but *Akkermansia* and *Parabacteroides* increased (**Figure 1C** **).** The operational taxonomic unit (OTU) count, Shannon-Weaver index, and Chao1 community richness all decreased in the ABX mice after 4 weeks of treatment (**Figure 1D**), indicating that antibiotic therapy altered the gut microbiota composition and decreased its abundance and diversity.

**Figure 2:**
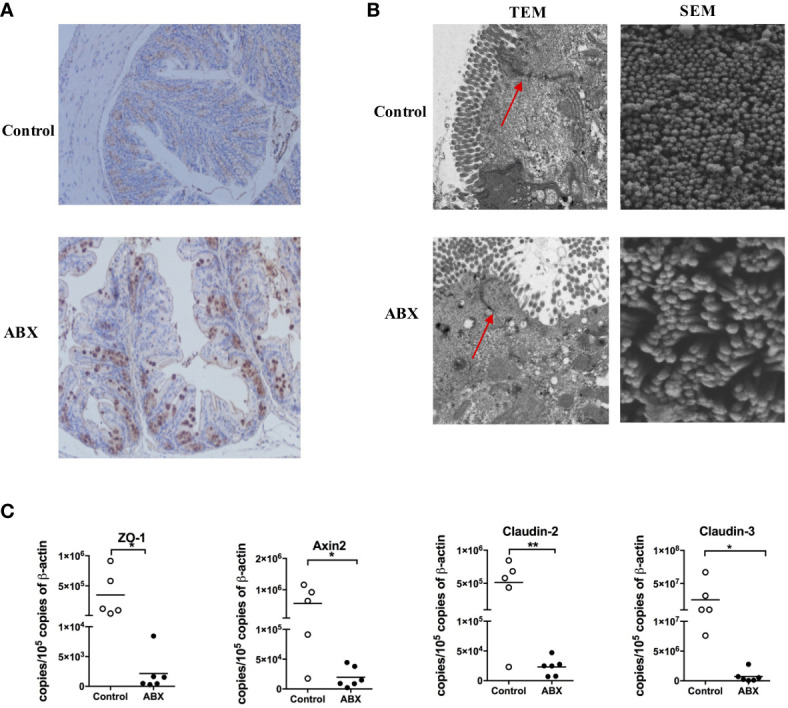
Gut microbiota depletion impaired the epithelial integrity of the colon. **(A)** IHC staining of *E*. *coli* in colon tissue sections of control and ABX mice after being fed with water or ABX for four weeks (magnification 200×). **(B)** The morphology of epithelia in the colon evaluated using TEM and SEM at 5000× and 60000× magnification, respectively. **(C)** The mRNA levels of molecules related to barrier function assayed *via* qPCR. n = 4-10/group, *P < 0.05, **P < 0.01.

ABX treatment enlarged the cecum, as described previously (8); however, no changes in the colon HE staining (**Supplementary Figure 1**). To validate microbial translocation in the colon, we stained paraffin-embedded colon tissue sections with a polyclonal antibody against *E. coli*. There was no *E. coli* deposition in the control colon tissue, however, numerous *E. coli* deposition was observed in the ABX-treated colon tissue (**Figure 2A**). The TEM image indicated swollen mitochondria and fragmented endoplasmic reticulum in the colon epithelia of ABX mice; while they were absent in the control mice. Meanwhile, the microvilli were shorter and were severely damaged in ABX mice. The tight junctions were also damaged and they became blurry (**Figure 2B**, TEM panels). In the SEM image, the microvilli were few and scattered, with an irregular architecture (**Figure 2B**, SEM panels). Meanwhile, ABX treatment inhibited the expression of the tight junction protein, zonula occludens-1 (ZO-1), and the molecules related to barrier function, including Axin2, Claudin-2, and Claudin-3 (**Figure 2C**).

### Commensal Bacteria Translocated to the Liver After Gut Microbiota Depletion

**Figure 3:**
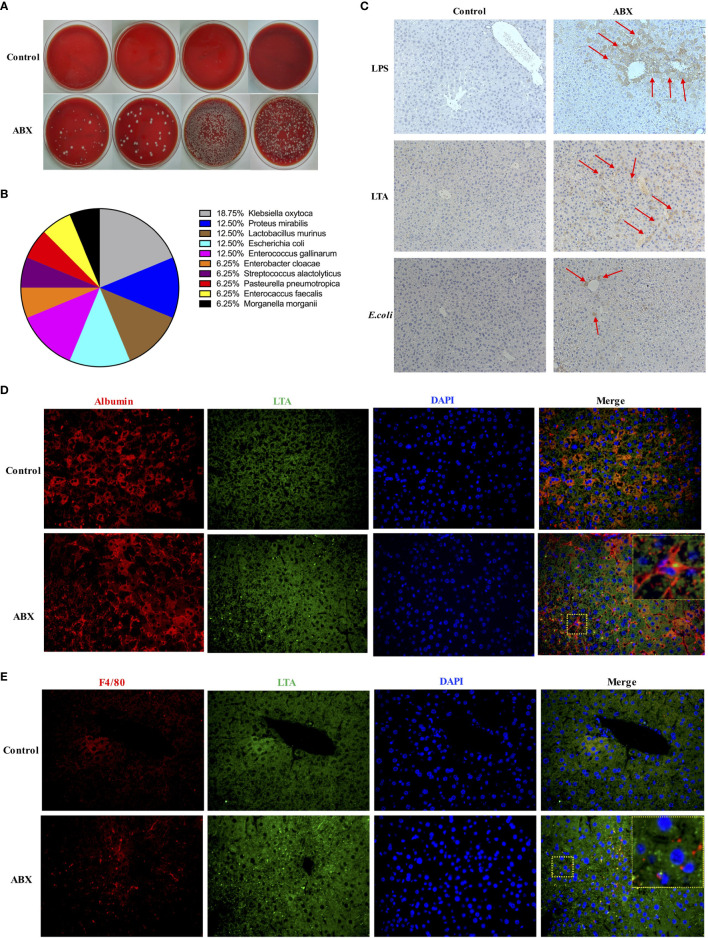
Commensal bacteria translocated to the liver after gut microbiota depletion. **(A)** Bacterial colonies after overnight cultivation of liver homogenates from control and ABX mice on blood agar plates. **(B)** Composition of 16 identified colonies. **(C)** LPS, LTA, and *E*. *coli* deposits in liver tissue section as measured *via* IHC staining (200×). Immunofluorescence microscopy images showing LTA (blue, 400×) deposited in hepatocytes **(D)** and Kupffer cells **(E)**. Nuclei (green), hepatocytes and Kupffer cells (red). The boxed area in the slide is enlarged and shown in the inset. n = 4-10/group.

To investigate whether gut commensal bacteria can translocate to the liver following gut microbiota depletion, the liver homogenates from control and ABX-treated mice were cultured in blood agar plates. Many bacterial colonies grew in the liver homogenate from ABX mice, but no bacterial colony was observed in the liver samples from the control mice (**Figure 3A**). Sixteen colonies from the liver homogenate of ABX mice were selected randomly and 10 genera were identified *via* mass spectrometry. These bacterial genera comprised the commensal microbiota in the gut of the mice (**Figure 3B**). For immunohistochemical analysis, the liver sections were stained with antibodies against lipopolysaccharide (LPS), lipoteichoic acid (LTA), and *E. coli*. Very few cells were positive for LPS, LTA, or *E. coli* in the liver samples from control mice. By contrast, we found strong signals for LPS and LTA and moderate signals for *E. coli* in the liver sections from ABX mice. LPS+ and *E. coli*+ cells were mainly located in the portal area, and LTA+ cells were observed in the peripheral area (**Figure 3C**). To observe the localization of the bacteria and the interaction among the cell types in the liver, sections were subjected to immunofluorescence staining and analyzed microscopically. LTA was deposited in both the albumin+ hepatocytes and F4/80+ Kupffer cells (**Figures 3D, E**), but LPS was not deposited in the hepatocytes and Kupffer cells (data not shown).

**Figure 4:**
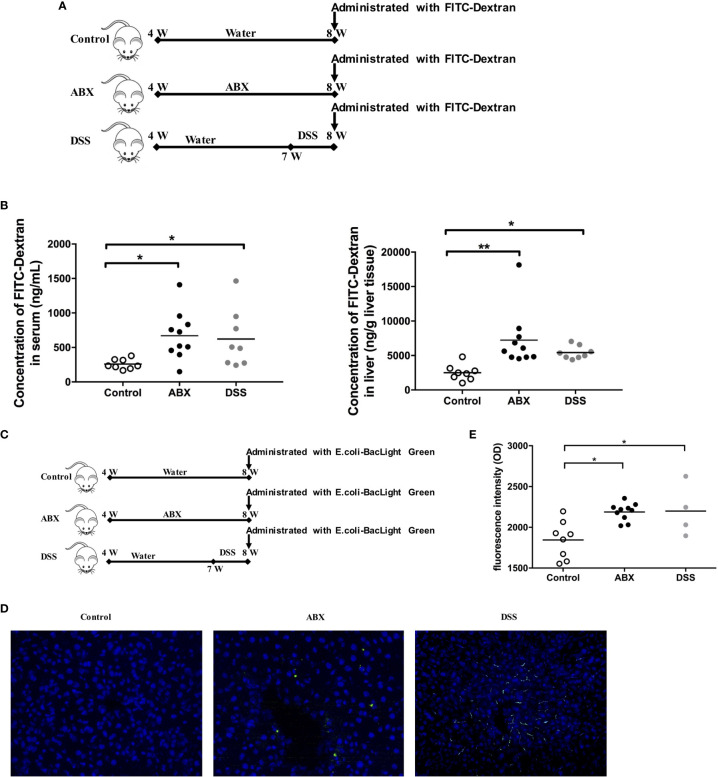
Evaluation of gut permeability and tracing of live bacterial translocation *in vivo*. **(A)** Schedule of the therapy. Mice were fed water or ABX from week 4 to week 8. For the positive control, mice were fed with water from week 4 to week 7, then switched to DSS till week 8. After termination of the therapy for 24 hours, mice were administered FITC-Dextran using oral gavage. **(B)** The concentrations of FITC-dextran in sera and liver homogenates 2 h after administration. **(C)** Schedule of the therapy. After termination of the therapy for 24 hours, mice were administrated with live BacLight Green-labeled *E*. *coli* using oral gavage. **(D)** The live *E*. *coli*-BacLight Green in liver tissue sections (400×) and **(E)** the fluorescence intensity in sera 4 h after administration. n=10/group, *P < 0.05, **P < 0.01.

To evaluate gut permeability after gut microbiota depletion, mice were fed water or ABX for 4 weeks. As a positive control, mice were fed with water for 3 weeks, and then treated with dextran sodium sulfate (DSS) to induce gut epithelial damage and colitis in the mice. After termination of the therapy for 24 hours, mice were administered 4.4 kDa FITC-dextran (**Figure 4A**). The intensities of FITC-dextran detected in the sera and liver homogenates of ABX mice were comparable to that in DSS-treated mice, but significantly higher than that in control mice (**Figure 4B**).

Furthermore, we traced live gut bacterial translocation to the liver *in vivo*. The BacLight Green Bacterial Stain for *E. coli* was administered to mice by oral gavage after the mouse model was established (**Figure 4C**). Liver sections were collected and were analyzed using fluorescence microscopy. BacLight Green-labeled *E. coli* were observed both in the liver of ABX and DSS mice, but not in control mice (**Figure 4D**). The fluorescence intensities were significantly higher in both ABX and DSS mouse sera than in the control mouse sera (**Figure 4E**). Taken together, depletion of gut microbiota by antibiotic treatment in this model impaired gut permeability and promoted gut microbiota translocation to the liver.

### Depletion of Gut Microbiota Impaired T Cell Function in the Liver

**Figure 5:**
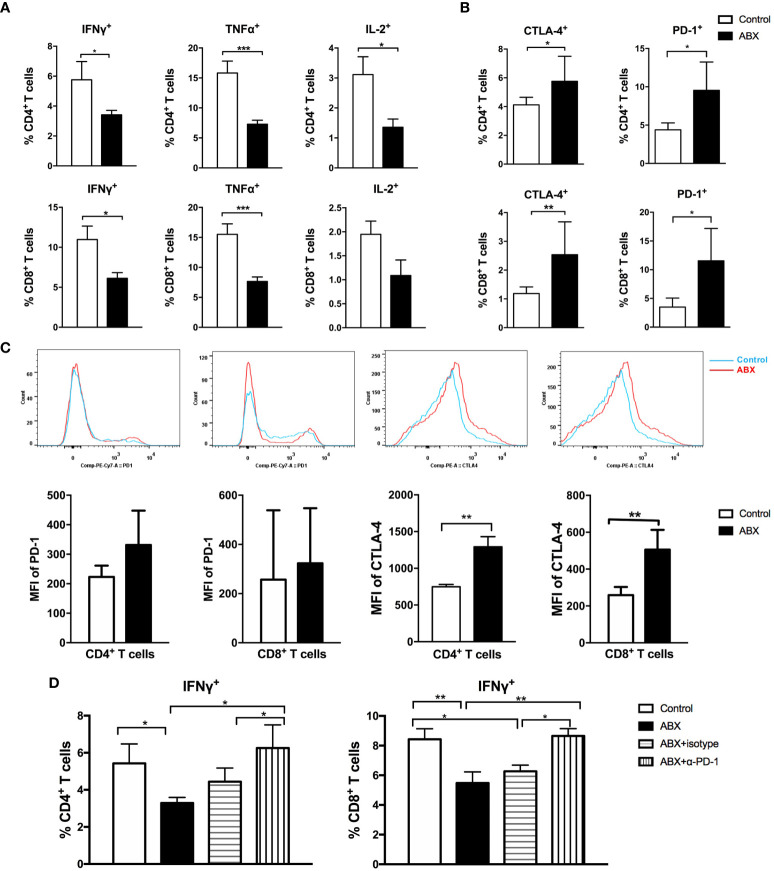
T cell function in liver was impaired after gut microbiota depletion. Intrahepatic lymphocytes were isolated and stimulated with anti-CD3 and anti-CD28. The expression of intracellular cytokines **(A)**, immune check point molecules PD-1 and CTLA-4 **(B)**, the MFI of PD-1 and CTLA-4 **(C)** in lymphocytes were analyzed. **(D)** After blocking with α-PD-1, the expression of IFN-γ in T cells were analyzed. n=10/group, *P < 0.05, **P < 0.01, ***P < 0.001.

To further evaluate the effect of gut microbiota depletion on liver immunity, we assayed the immune cell composition and function in mice liver. After 4 weeks of feeding with water or ABX, no significant difference in the frequencies of CD4+ and CD8+ T cells was observed between the livers of control and ABX mice **(** **Supplementary Figure 2A** **)**. Two populations of essential immunosuppressive cells, regulatory T cells (Tregs) and myeloid-derived suppressor cells (MDSCs), were also comparable between the livers of control and ABX mice **(** **Supplementary Figure 2B** **)**. After stimulation with anti-CD3 and anti-CD28, CD4+ T cells from the liver of ABX mice produced significantly lower IFN-γ, TNF-α, and IL-2 levels than control mice. The production of IFN-γ and TNF-α in CD8+ T cells from ABX mice also decreased significantly. The production of IL-2 in CD8+ T cells from ABX mice showed a decreasing trend, although no significant effect was observed (**Figure 5A**). CD107a expression in CD4+ and CD8+ T cells was comparable in the control and ABX mice **(** **Supplementary Figure 2C** **)**.

To explore the molecular mechanisms underlying impairment of T cell function following gut microbiota depletion, the expression of immunosuppressive molecules was investigated. The frequencies of PD-1+ and CTLA-4+ cells increased significantly among CD4+ and CD8+ T cells from ABX mice (**Figure 5B**). Among CD8+ T cells, the frequencies of Tim-3+ and LAG-3+ cells increased significantly in ABX mice **(** **Supplementary Figure 2D** **)**. The mean fluorescence intensity (MFI) of PD-1 in CD4+ and CD8+ T cells was comparable between the control and ABX mice, but the MFI of CTLA-4 increased significantly in CD4+ and CD8+ T cells of ABX mice (**Figure 5C**).

*In vitro*, PD-1 blockade enhanced IFN-γ production in CD4+ and CD8+ T cells from ABX mice and restored it to the levels in control mice (**Figure 5D**), but TNF-α and IL-2 expression did not change significantly after PD-1 blockade **(** **Supplementary Figure 2E** **)**. *In vitro* CTLA-4, Tim-3, and LAG-3 blockade had no effect on the restoration of cytokine production in both CD4+ and CD8+ T cells from ABX mice (data not shown). These results suggest the existence of other potential mechanisms for T cell functional impairment after gut microbiota depletion.

### Depletion of Gut Microbiota Prolonged HBV Infection in Mice

**Figure 6:**
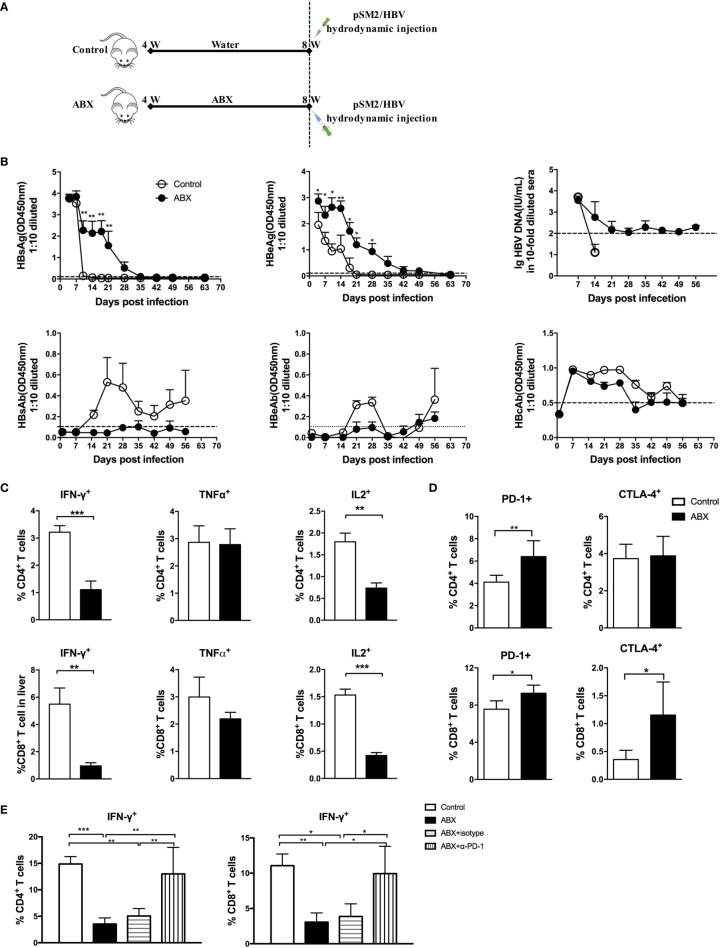
Gut microbiota depletion impaired antiviral immune response and prolonged HBV infection. **(A)** Schedule of the experiment. Mice were fed with water or ABX for 4 weeks and then hydrodynamically injected with pSM2/HBV. **(B)** HBsAg, HBeAg, HBsAb, HBeAb, HBcAb, and HBV DNA levels in sera were monitored at the indicated times. HBV DNA can’t be detected after 14 days in control group and no data was shown. The cutoff values are shown as dotted lines. Mice were euthanized on day 12 after HBV infection; intrahepatic lymphocytes were stimulated with HBV core epitope and anti-CD28. The expression of intracellular cytokines **(C)**, immune checkpoint molecules PD-1 and CTLA-4 **(D)** were analyzed. **(E)** After blocking with α-PD-1, the expression of IFN-γ in T cells was analyzed. n=10/group, *P < 0.05, **P < 0.01, ***P < 0.001.

To evaluate the role of gut microbiota depletion in the outcomes of HBV infection, mice were hydrodynamically injected with pSM2/HBV to establish a transient HBV infection model (**Figure 6A**). Hepatitis B surface antigen (HBsAg) in the sera of control mice was cleared within 14 days after hydrodynamic injection (HI), but persisted for up to 35 days in ABX mice. Hepatitis B e antigen (HBeAg) in control mice was cleared within 21 days after HI, but persisted for more than 49 days in ABX mice, with a higher HBeAg titer from day 1 to day 28 after HI. HBV DNA became undetectable in the control mice after 14 days, but was detected with a low titer in ABX mice for 56 days. Several mice developed hepatitis B surface antibodies (HBsAb), hepatitis B e antibodies (HBeAb), and hepatitis B core antibodies (HBcAb) in both control and ABX mice, but no significant difference was observed (**Figure 6B**).

HBV-specific T cell response was assayed *via* fluorescence-activated cell sorting (FACS) after stimulation with an H2-K^b^ restricted CD8+ peptide which derived from HBcAg as previously described (2, 14). Both CD4+ and CD8+ T cells response were observed after the peptide stimulation, which could be due to the cross reaction of the peptide (15). IFN-γ and IL-2 production decreased significantly in both HBV-specific CD4+ and CD8+ T cells from ABX mice. TNF-α production in HBV-specific CD4+ and CD8+ T cells was comparable in control and ABX mice (**Figure 6C**). After HI with HBV plasmid, PD-1 expression was enhanced in CD4+ and CD8+ T cells from ABX mice, whereas CTLA-4 expression was only enhanced in CD8+ T cells from ABX mice (**Figure 6D**). *In vitro* blockade of PD-1 restored IFN-γ production in HBV-specific CD4+ and CD8+ T cells in ABX mice to the levels in control mice (**Figure 6E**), but TNF-α and IL-2 expression remained unchanged after PD-1 blockade (data not shown). Additionally, *in vitro* blocking of CTLA−4 had no significant effect on cytokine production (data not shown).

## Discussion

In this study, we used broad-spectrum antibiotics to deplete the gut microbiota in mice and found that: 1) gut microbiota depletion induced by ABX is associated with damage of colon epithelial integrity; 2) live commensal gut microbiota can translocate to the liver; 3) gut microbiota depletion induced by ABX treatment can lead to impaired T cell function in the liver and hamper the immune clearance of HBV infection; and 4) impaired T cell function in the liver partially relies on enhanced PD-1 expression.

Increasing evidence indicates that the composition of gut microbiota plays an essential role in immune system development, which in turn modulates gut barrier function (16). Gut microbiota dysbiosis is associated with increasing gut permeability in both intestinal and extra-intestinal disorders (17). In this study, commensal bacteria were found in the lamina propria and liver, the commensal bacterial components, LPS and LTA, deposited in liver, and live bacteria transferring from the gut to the liver were monitored. This indicated that gut microbiota depletion induced by antibiotic therapy impaired gut barrier function. Although electron microscopy confirmed colon epithelial cell injuries, it remains unclear how alteration in the gut microbiota causes this damage and disrupts gut barrier function. Previous studies have indicated that commensal microbes play an essential role in determining gut epithelial cell turnover rates and regeneration (18, 19). Short chain fatty acids (SCFAs) produced by the gut microbiota serve as nutrient supplements that modulate intestinal barrier function (16, 20, 21).

Maintenance of the epithelial barrier is associated with tight structures, which depend on the expression of tight junction proteins, molecules related to barrier function, and the mucus layer. Commensal bacterial colonization has been reported to regulate the expression of molecules related to tight structures (6), and several *Lactobacillus* stains can stabilize tight junctional structures after intestinal epithelial impairment (22, 23). In this study, we observed for the first time that the tight junction protein (ZO-1) and molecules related to barrier function (Axin2, Claudin-2, and Claudin-3) were downregulated following gut microbiota depletion through antibiotic treatment. AMP-activated protein kinase (AMPK), a crucial regulator of energy homeostasis, can regulate epithelial barrier function by promoting the assembly of tight junctions *via* direct protein phosphorylation related to tight structures (24). AMPK activation can be influenced by commensal bacterial colonization (25). SCFAs produced by the gut microbiota can also enhance the intestinal barrier by promoting the assembly of tight junction though AMPK activation (26). Whether the expression of proteins related to tight junction structures depends on the AMPK activation induced by the gut microbiota still requires further study.

Increasing evidence indicates that live commensal bacteria can translocate to sterile organs under pathogenic conditions (5–7). Bacterial LPS and LTA have been detected in multiple cancer types, and live metabolically active bacteria have been found in freshly resected human breast tumors (27). Most of the bacteria in tumor tissue are intracellular and localize in both cancer and immune cells (27). Previous studies have indicated that depletion of gut microbiota through antibiotic therapy can lead to the translocation of live commensal bacteria from the colon to the MLN and induce an inflammatory response (5). In our study, we extended this perspective and confirmed that depletion of gut microbiota by antibiotic therapy can induce live commensal bacterial translocation from the gut to the liver. It is worth mentioning that the translocation of live bacteria from the gut to other sterile organs did not induce sepsis in the above studies (5–7). The intestinal dendritic cells (DC) can capture the live commensal bacteria and traffic to the MLN, and clear the invading pathogens with the cooperation of other immune cells (28). Kupffer cells can also capture and clear the invasive commensal bacteria in the liver (29). Indeed, the bacteria captured by intestinal DC or Kupffer cells do not normally penetrate further to reach systemic secondary lymphoid structures. The MLN and liver can act as the first and second firewalls, respectively, between the gut microbiota and venous blood circulation (28, 29).

HBV infection can develop to either acute or chronic infection, which depends on the immune response of the host. Acute HBV infection can induce higher frequencies, multi-specific T cells. By contrast, specific T cells exhibit lower frequencies and functional exhausted response in chronic HBV infection. Mice can’t be infected by HBV directly due to the species specificity of HBV. Hydrodynamic injection of plasmid pSM2/HBV can induce transient HBV replication in the liver of mice and mimics the acute HBV infection (2). And plasmid pAAV/HBV1.2 (2) or recombinant rAAV8/HBV1.3 (30, 31) can induce persistent HBV replication, which can be used to establish chronic HBV infection model. In this study, we used the acute HBV infection mice model and found that gut microbiota depletion could impair antiviral immunity in the liver, thereby delaying HBV infection clearance. Translocated bacteria and their components play an essential role in modulating HBV-specific immune response. LPS and LTA, cell wall components from Gram-negative and Gram-positive bacteria, respectively, were found in the liver after ABX treatment. LPS is a toll like receptor 4 (TLR4) ligand and can promote liver sinusoidal endothelial cells and Kupffer cells to enhance IL-10 and TGF-β production, which in turn induces liver immune tolerance (32, 33). LTA is a TLR2 ligand, and can induce IL-10 production in Kupffer cells through MAPK pathway activation (34). In an obesity-associated liver cancer model, LTA in the liver enhanced the senescence-associated secretory phenotype of hepatic stellate cells (HSCs) and upregulated COX2 expression and prostaglandin E_2_ (PGE_2_) production in the senescent HSCs, which in turn suppressed antitumor immune response through the PGE_2_ receptor on immune cells (35). It’s worth to mention that some antibiotics can elicit immune cell response, independent of microbiota dysbiosis, by inhibiting the respiratory activity (36), or blocking the mitochondrial protein synthesis in immune cells (37). The antibiotics cocktail in this study was widely used to investigate the role of the gut microbiota in modulation of host immune response, and the side effects were rarely considered (5, 6, 8–10). Whether this antibiotics cocktail can impair T cells function or gut barrier function directly should be further studied.

The upregulation of immune check point molecules observed in this study also played a critical role in suppressed HBV-specific T cell response. Although the expression of PD-1, CTLA-4, Tim-3, and LAG-3 increased in CD4+ and/or CD8+ T cells, only PD−1 partially contributed to the impairment of T cells function in the liver after gut microbiota depletion. Previous studies have indicated that LPS induces PD-1 expression in macrophages *via* the NF-κB pathway (38) and accumulates cyclic AMP to enhance CTLA-4 production in T cells (39); however, it remains unclear how the gut microbiota regulates the expression of immune check point molecules in T cells in the liver. It is worth mentioning that the immune checkpoint blockade therapy depends on the gut microbiota composition. Between responders versus non-responders among melanoma patients receiving anti-PD-1 therapy, gut microbiota composition differed significantly (40, 41). Gut microbiota dysbiosis induced by antibiotics is related to the failure of PD-1 and PD-L1 therapy in patients with non-small cell lung cancer and renal cell carcinoma (42). The interaction of gut microbiota and immune check point molecules should be further studied.

Taken together, our study demonstrated that depletion of gut microbiota by broad-spectrum antibiotic impaired gut barrier function, increased gut permeability, and promoted commensal bacterial translocation from the gut to the liver, leading to suppressed T cell response in the liver and prolonged HBV infection. Thus, our data indicate the protective role of the gut microbiota in gut barrier function and T cell antiviral response in the liver. These findings are potentially applicable when determining patient-specific HBV therapy and liver disease treatment.

## Data Availability Statement

The raw data supporting the conclusions of this article will be made available by the authors, without undue reservation.

## Ethics Statement

The animal study was reviewed and approved by Institutional Animal Care and Use Committee at Tongji Medical College.

## Author Contributions

JW and BW designed the study, analyzed data, and wrote the paper. WG, XZho, and XL performed experiments and analyzed data. QZ performed partial experiments. BZ and JP collected samples and data. XZhe, YL, and DY analyzed data. All authors contributed to the article and approved the submitted version.

## Funding

This work was supported by the National Science and Technology Major Project for Infectious Diseases of China (2017ZX10304402-002-005), the Chinese National Key Technology R&D Program (2015BAI09B06), and National Natural Science Foundation of China (grant numbers 81501748, 91642118, 81461130019). The funders had no role in the study design, data collection and analysis, decision to publish, or preparation of the manuscript.

## Conflict of Interest

The authors declare that the research was conducted in the absence of any commercial or financial relationships that could be construed as a potential conflict of interest.

## Abbreviations

ABX, broad-spectrum antibiotics; AMPK, AMP-activated protein kinase; CFU, colony-forming unit; DC, dendritic cells; DSS, dextran sodium sulfate; FACS, fluorescence-activated cell sorting; HBV, hepatitis B virus; HBsAg, hepatitis B surface antigen; HBeAg, hepatitis B e antigen; HBsAb, hepatitis B surface antibody; HBeAb, hepatitis B e antibody; HBcAb, hepatitis B core antibody; HI, hydrodynamic injection; HSCs, hepatic stellate cells; MALDI-TOF, matrix-assisted laser desorption/ionization time-of-flight; MDSC, myeloid-derived suppressor cells; MIB, mouse intestinal Bacteroides; MLN, mesenteric lymph node; MFI, mean fluorescence intensity; LPS, lipopolysaccharide; LTA, lipoteichoic acid; OUT, operational taxonomic unit; PGE_2_, prostaglandin E_2_; SCFAs, short chain fatty acids; SEM, scanning electron microscopy; SPF, specific pathogen-free; TEM, transmission electron microscopy; TLR, toll like receptor; Tregs, regulatory T cells; ZO-1, zonula occludens-1.
