# Supplementary material for: Depletion of Gut Microbiota Impairs Gut Barrier Function and Antiviral Immune Defense in the Liver
Source: Front Immunol. 2021 Mar 25;12:636803. doi: 10.3389/fimmu.2021.636803 (PMC8027085; doi:10.3389/fimmu.2021.636803)

Supplementary information

Material and Methods

Immunohistochemistry (IHC) and immunofluorescence analysis

IHC and immunofluorescence staining were performed as previously described(1). Colon and liver samples were fixed in 4% paraformaldehyde and embedded in paraffin. For IHC, the sections were stained with anti-lipoteichoic acid (LTA), anti-lipopolysaccharide (LPS) core, or anti-*Escherichia coli* antibody. For immunofluorescence, the sections were stained with respective primary antibodies at 4°C overnight. After washing, the sections were incubated with the respective secondary antibodies for 4 h at room temperature. Immunofluorescence microscopy was performed using an Olympus microscope.

The following antibodies were used: mouse anti-LTA monoclonal (1:50; Thermo Fisher Scientific, USA), mouse anti-LPS core monoclonal (1:100; Abbexa Ltd, UK), and rabbit anti-*E. coli* (1:500; DAKO, Denmark), rat anti-F4/80 monoclonal (1:100; Abcam, UK), rabbit anti-albumin polyclonal (1:100; GeneTex, USA), Cy3-conjugated goat anti-rabbit (1:100; Boster, China), fluorescein isothiocyanate (FITC)-conjugated goat anti-mouse (1:100; Boster), and Cy3-conjugated goat anti-rat (1:100; Boster). DAPI (Beyotime, China) was used for nuclei staining.

Lymphocyte isolation and flow cytometry

Intrahepatic lymphocytes were isolated as described in a previous study(2). The lymphocytes were stimulated with 1 μg/ml anti-CD3 antibody (eBioscience, San Diego, CA, USA) or CD8+ T cell epitope (K^b-HBV Cor93–100 epitope, MGLKFRQL, 10 μg/mL) at 37°C for 5 h in the presence of 1 μg/mL anti-CD28 antibody (eBioscience). For the PD-1 and CTLA-4 blockade *in vitro*, 10 μg/mL αPD-1 (clone 29F.1A12; Biolegend, USA), 10 μg/mL αCTLA-4 (clone 9H10; Biolegend), or 20 μg/ml αTim-3 (clone RMT3-23; Biolegend) was added to the RPMI1640 medium and cultured for 3 days as previously described(3,4). The lymphocytes were cultured with Rat IgG2a κ Isotype Ctrl Antibody (clone RTK2758; Biolegend) or Syrian Hamster IgG Isotype Ctrl Antibody (clone SHG-1; Biolegend) as control. Antibodies used for surface staining were BV421-anti-CD8, APC-Cy7-anti-CD4, PE-Cy7-anti-CD107a, FITC-anti-Gr1, APC-Cy7-anti-CD11b, FITC-anti-Tim3, PE-anti-CTLA4, PerCP-Cy5.5-anti-LAG3, and PE-Cy7-anti-PD1 (eBioscience). For intracellular cytokine staining, the antibodies used were APC-anti-IFN-γ, PerCP-Cy5.5-anti-IL-2, and FITC-anti-TNF-α (Biolegend). All the samples were stained with Fixable Viability Dye eFluor 506 (eBioscience) to exclude dead cells and analyzed using a BD FACSCanto II flow cytometer. Data were analyzed using FlowJo software (Tree Star, Ashland, OR, USA).

Supplementary Figure Legends

Supplementary Fig. 1. Effect of broad-spectrum antibiotics (ABX) therapy on the cecum and colon.

Representative images (200×) of ceca (A) and HE staining of the colon (B) from control and ABX-treated mice after a 4-week therapy. N = 4–10/group.

Supplementary Fig. 2. Intrahepatic lymphocyte phenotypes after gut microbiota depletion.

The frequencies of CD4+ and CD8+ T cells (A), Treg cells and myeloid-derived suppressor cells (MDSC, defined as CD11b+Gr1+) (B) in intrahepatic lymphocytes from mice were analyzed using fluorescence-activated cell sorting after feeding with water or broad-spectrum antibiotics (ABX) for 4 weeks. (C) The expression of 107a in intrahepatic lymphocytes stimulated with anti-CD3 and anti-CD28 antibody. (D) The frequencies of Tim-3+ and LAG-3+ in intrahepatic lymphocytes; (E) TNF- α and IL-2 expression in T cells after blocking the immune checkpoint with α-PD-1. n = 10/group, *P < 0.05, **P < 0.01.

References

1. Estes JD, Harris LD, Klatt NR, Tabb B, Pittaluga S, Paiardini M, et al. Damaged intestinal epithelial integrity linked to microbial translocation in pathogenic simian immunodeficiency virus infections. *PLoS Pathog* (2010) 6(8):e1001052. doi:10.1371/journal.ppat.1001052.

2. Zhu Q, Xia P, Zhou X, Li X, Guo W, Zhu B, et al. Hepatitis B Virus Infection Alters Gut Microbiota Composition in Mice. *Front Cell Infect Microbiol* (2019) 9:377. doi:10.3389/fcimb.2019.00377.
3. Duraiswamy J, Kaluza KM, Freeman GJ, and Coukos G. Dual blockade of PD-1 and CTLA-4 combined with tumor vaccine effectively restores T-cell rejection function in tumors. *Cancer Res* (2013) 73(12):3591-3603. doi:10.1158/0008-5472.CAN-12-4100.
4. Nakae S, Iikura M, Suto H, Akiba H, Umetsu DT, Dekruyff RH, et al. TIM-1 and TIM-3 enhancement of Th2 cytokine production by mast cells. *Blood* (2007) 110(7):2565-2568. doi:10.1182/blood-2006-11-058800.

Supplementary Fig 1

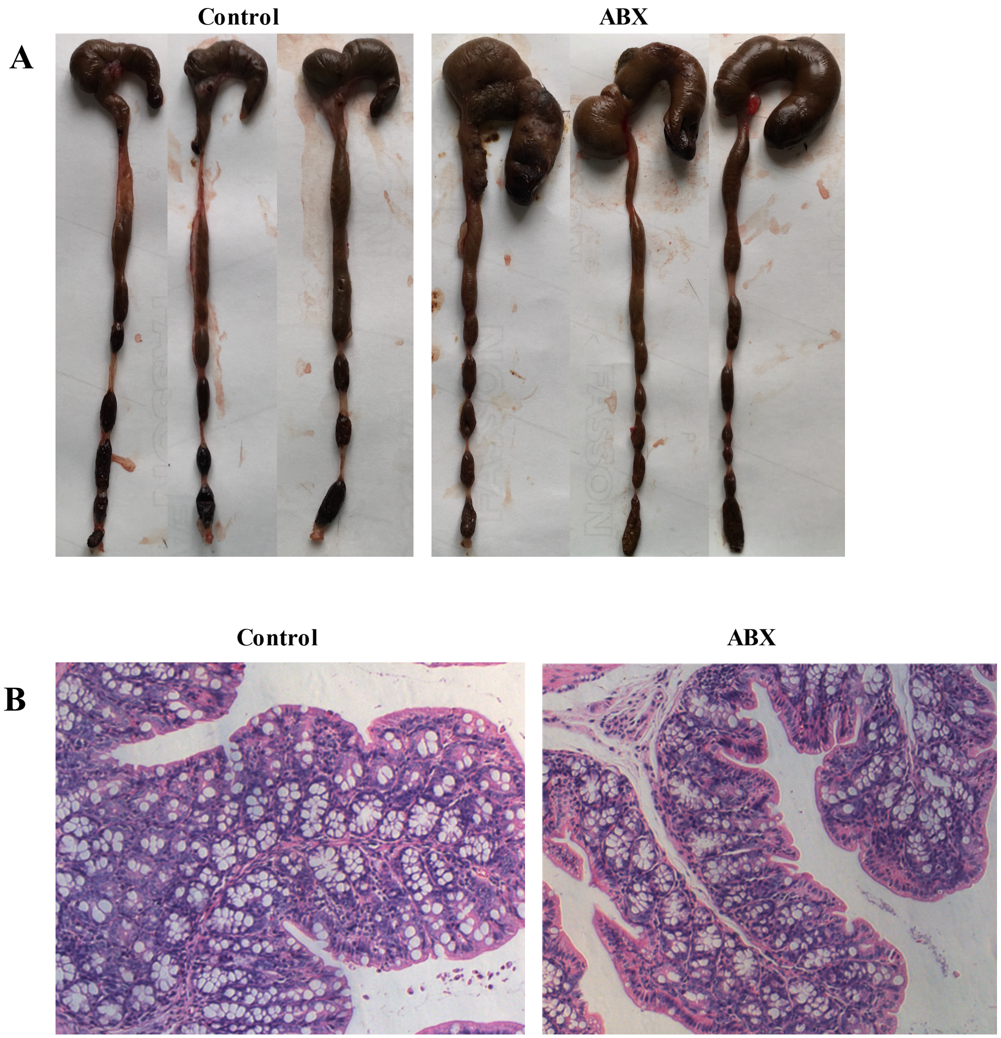

Supplementary Fig 2

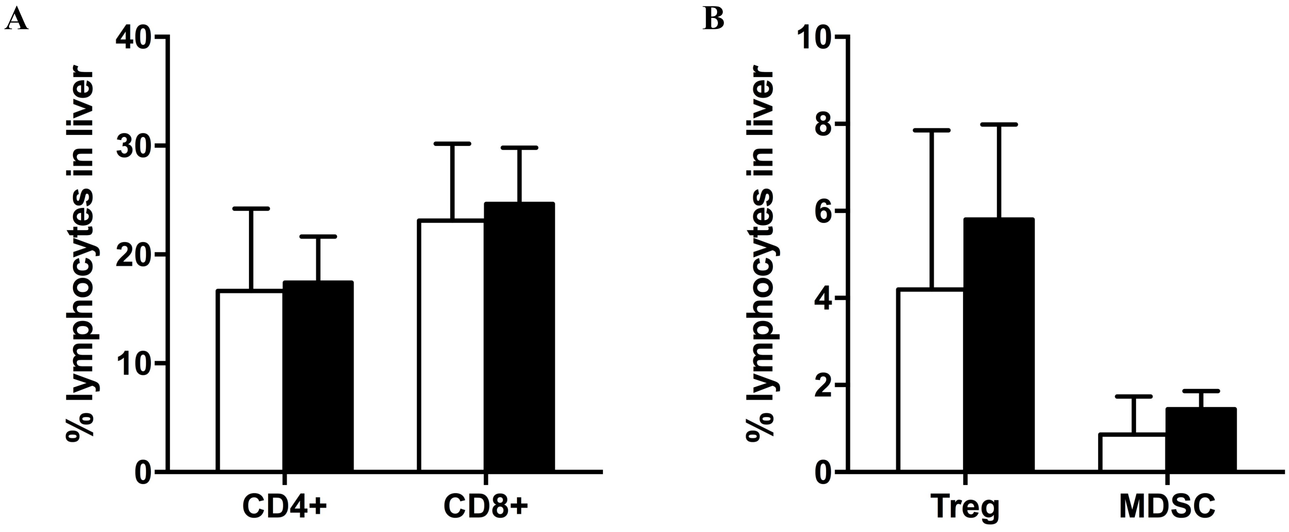

Supplementary Fig 2

C

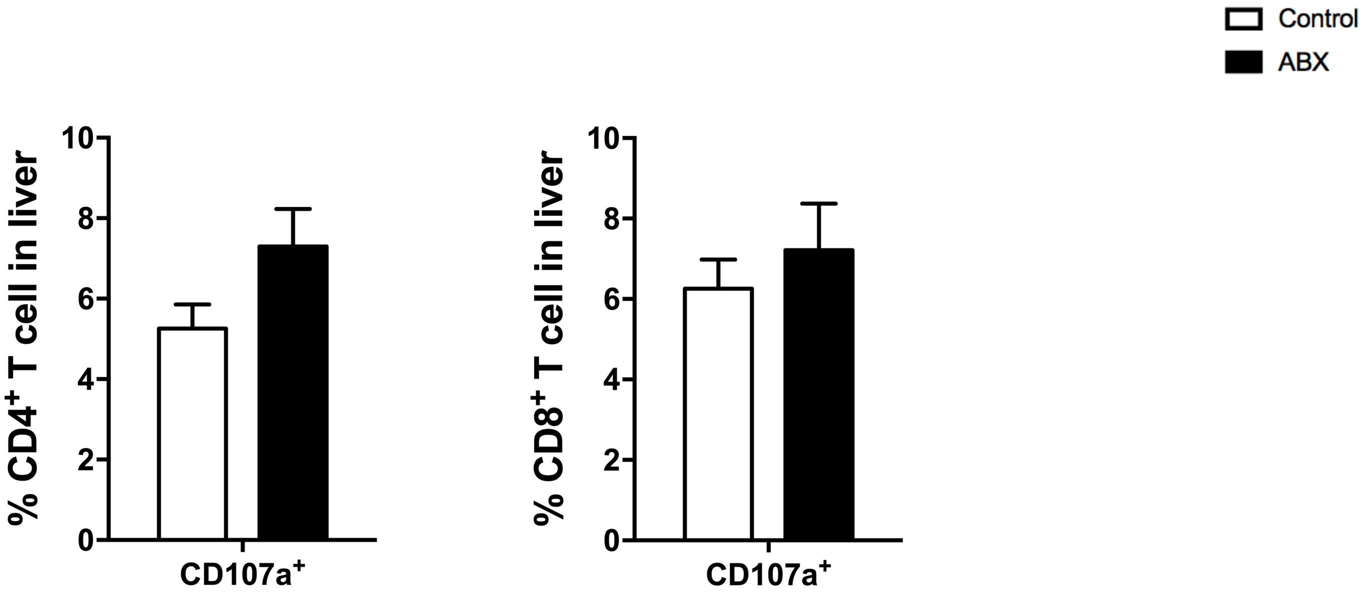

Supplementary Fig 2

D

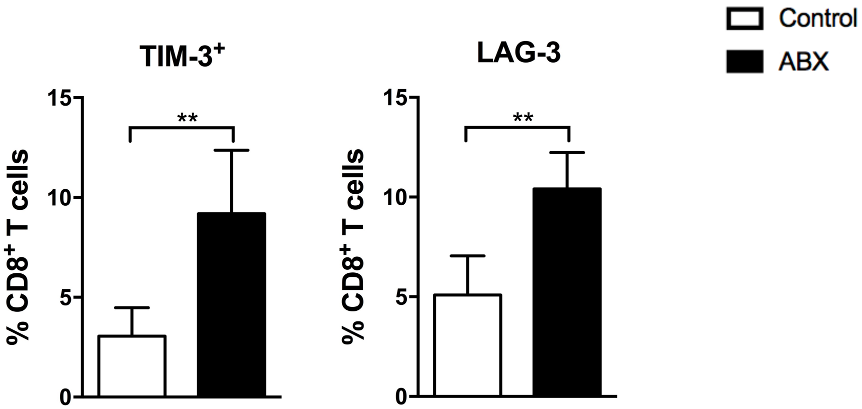

Supplementary Fig 2

E

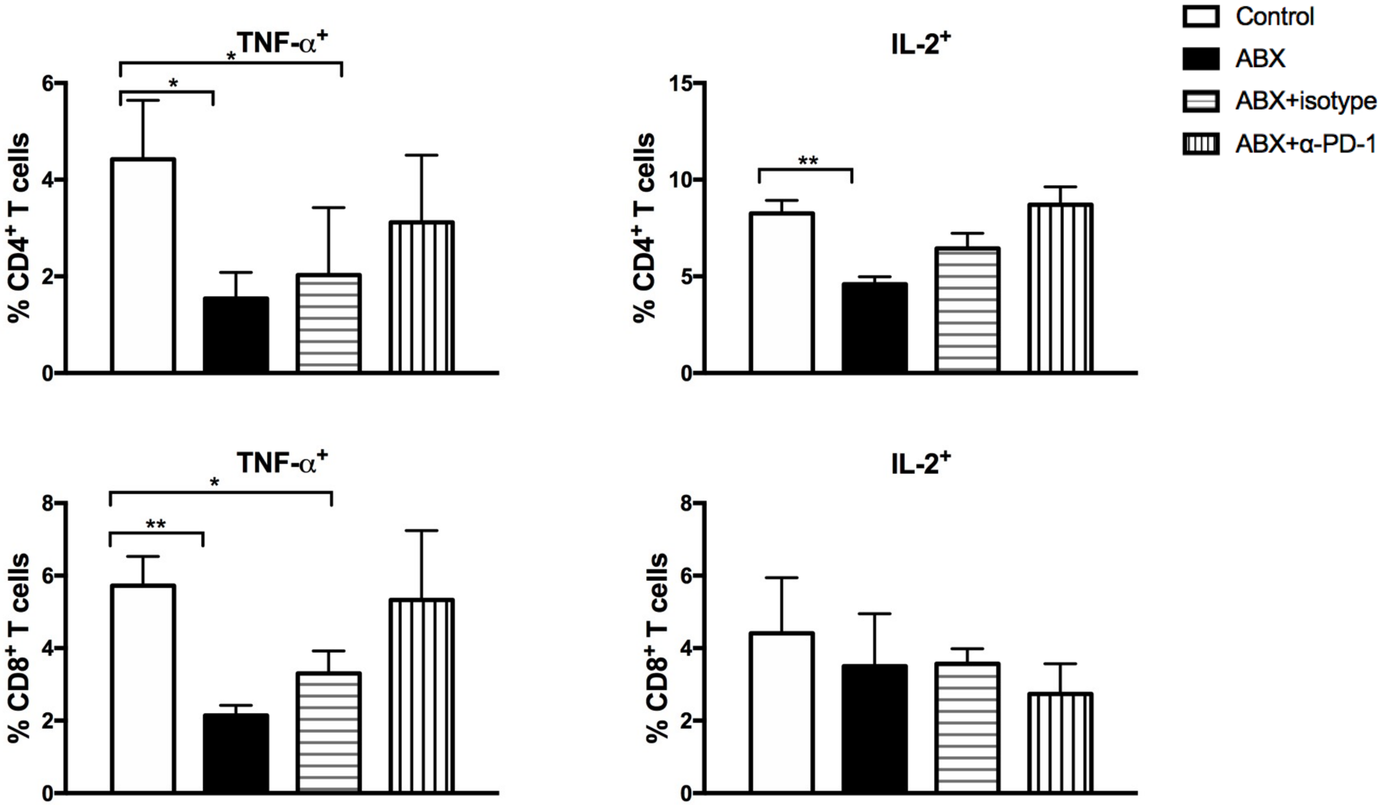

Supplement: Supplementary file 1 [file DataSheet_1.pdf]
